# A meta-analysis of risk factors for non-superficial surgical site infection following spinal surgery

**DOI:** 10.1186/s12893-023-02026-2

**Published:** 2023-05-16

**Authors:** Xiaowen Liu, Yang Hou, Hongyang Shi, Tianyi Zhao, Haoyang Shi, Jiangang Shi, Guodong Shi

**Affiliations:** Department of Orthopaedic Surgery, Changzheng Hospital, Second Military Medical University, No. 415 Fengyang Rd, Shanghai, 200003 China

**Keywords:** Spinal surgery, Risk factors, Deep surgical site infection, Organ/space surgical site infection, Non-superficial surgical site infection

## Abstract

**Background:**

Surgical site infection (SSI) is the most common complications in spinal surgery. In SSI, non-superficial surgical site infections are more likely to result in poor clinical outcomes. It has been reported that there are multiple factors contributing to postoperative non-superficial SSI, but still remains controversial. Therefore, the aim of this meta-analysis is to investigate the potential risk factors for non-superficial SSI following spinal surgery.

**Methods:**

A systematic database search of PubMed, Embase, Web of Science, Cochrane Library and Clinical Trials was performed for relevant articles published until September 2022. According to the inclusion and exclusion criteria, two evaluators independently conducted literature screening, data extraction and quality evaluation of the obtained literature. The Newcastle–Ottawa Scale (NOS) score was used for quality evaluation, and meta-analysis was performed by STATA 14.0 software.

**Results:**

A total of 3660 relevant articles were initially identified and 11 articles were finally included in this study for data extraction and meta-analysis. The results of meta-analysis showed that the diabetes mellitus, obesity, using steroids, drainage time and operative time were related to the non-superficial SSI. The OR values (95%CI) of these five factors were 1.527 (1.196, 1.949); 1.314 (1.128, 1.532); 1.687(1.317, 2.162); 1.531(1.313, 1.786) and 4.255(2.612, 6.932) respectively.

**Conclusions:**

Diabetes mellitus, obesity, using steroids, drainage time and operative time are the current risk factors for non-superficial SSI following spinal surgery. In this study, operative time is the most important risk factor resulting in postoperative SSI.

**Supplementary Information:**

The online version contains supplementary material available at 10.1186/s12893-023-02026-2.

## Introduction

SSI is a common complication after spinal surgery [1, 2]. In previous studies, postoperative SSI occurs in approximately 1–15% of patients who undergo spinal surgery [3, 4]. This postoperative complication could not only lead to poor prognosis of patients, but also increase medical costs [5, 6]. The Centers for Disease Control and Prevention (CDC) is responsible for monitoring SSI. In their previous report, the incidence of SSI associated with spinal fusion was estimated to be 4.15% [7]. However, there are few reports on the risk factors of postoperative deep surgical site infections(d-SSI) and organ/space SSI. As the type of SSI, d-SSI and organ/space SSI lead to more serious clinical outcomes, such as fever(> 38℃), pneumonia, localized pain, or tenderness, abscess and toxemia [8, 9]. To identify patient characteristics and perioperative risk factors associated with postoperative non-superficial SSI, we conduce this meta-analysis.

## Materials and methods

### Study selection

We conducted a systematic search of the scientific literature on non-superficial and performed a meta-analysis of the pooled data from the eligible studies. Case–control studies or cohort studies were searched from PUBMED, EMBASE, Web of Science, Cochrane Library and Clinical Trials independently by two authors. We adhered to the Preferred Reporting Items for Systematic Reviews and Meta-Analyses (PRISMA) and Meta-Analysis of Observational Studies in Epidemiology (MOOSE) guidelines. Taking PubMed as an example, the brief retrieval strategy is shown as follows: ((spin*[Title/Abstract]) AND (infections[Title/Abstract]) AND (risk factors[Title/Abstract]). Two evaluators independently screened the literature by adopting the unified inclusion criteria. In case of any disagreement, it was resolved through discussion or with the assistance of a third researcher.

### Inclusion criteria and exclusion criteria

The eligibility criteria were specified using the Population, Intervention, Criteria, Outcome and Study design (PICOS) framework. The selected literatures must meet the following conditions: 1) thoracolumbar or sacral spinal surgery; 2) The literature should adopt the CDC, ICD-9 or Horan et Al.’s definition of SSI; 3) The original data should provide OR value and 95% confidence interval (95%CI) or the OR value and 95%CI can be calculated from the data; 4) The summary results can be expressed by corresponding statistical indicators.

Literatures meeting one of the following conditions were excluded: (1) cervical spine surgery; (2) pediatric spinal surgery; (3) superficial surgical site infections; (4) The type of SSI is not clearly indicated; (5) animal studies; (6) meta-analysis and reviews; (7) duplicate studies; (8) case reports; (9) articles without available data; (10) unrelated studies.

### Definition of SSI

SSIs were defined according to the Centre for Disease Control and Prevention. The CDC definition categorizes SSI based on the layer of tissue involved, timeframe and the presence of implants [10].

According to the definition of SSI in CDC, a deep SSI occurs in the deep connective tissue (for example, fascial and muscle layers), within 30 days if there is no implant/instrumentation, or 1 year if an implant is in situ. patient has at least one of the following: a. purulent drainage from the deep incision. b. a deep incision that spontaneously dehisces, or is deliberately opened or aspirated by a surgeon, physician* or physician designee and organism(s) identified from the deep soft tissues of the incision by a culture or non-culture based microbiologic testing method which is performed for purposes of clinical diagnosis or treatment (for example, not Active Surveillance Culture/Testing (ASC/AST)) or culture or non-culture based microbiologic testing method is not performed. A culture or non-culture based test from the deep soft tissues of the incision that has a negative finding does not meet this criterion. and patient has at least one of the following signs or symptoms: fever (> 38 °C); localized pain or tenderness. c. an abscess or other evidence of infection involving the deep incision that is detected on gross anatomical or histopathologic exam, or imaging test [11–13].

An organ/space SSI involves any part of the body deeper than the fascial/muscle layers that is opened or manipulated during the operative procedure; patient has at least one of the following: a. purulent drainage from a drain that is placed into the organ/space (for example, closed suction drainage system, open drain, T-tube drain, CT-guided drainage). b. organism(s) identified from fluid or tissue in the organ/space by a culture or non-culture based microbiologic testing method which is performed for purposes of clinical diagnosis or treatment (for example, not Active Surveillance Culture/Testing (ASC/AST)). c. an abscess or other evidence of infection involving the organ/space that is detected on gross anatomical or histopathologic exam, or imaging test evidence suggestive of infection [11–13].

In the current study, deep surgical site infection and organ/space SSI are defined as the non-superficial SSI.

### Methodological quality evaluation

The Newcastle–Ottawa Scale (NOS) scoring system was used to evaluate the methodological quality of the included studies. For case–control studies, the NOS uses a "star" rating system to judge quality based on three factors: (a) selection; (b) comparability; (c) exposure. Among these three categories, studies can receive a maximum of 4, 2, and 3, with 9 stars being the highest rating. In the NOS, we assigned scores of 0–3, 4–6 and 7–9 to indicate low, moderate and high quality studies, respectively. Most included studies had moderate and high quality score. In this article, there are one article with 5 stars, five articles with 6 stars, three articles with 7 stars, and two articles with 8 stars, which is shown in the Table 1.

**Table 1:** Newcastle–Ottawa quality assessment of the studies included in the systematic review

Study	Quality Assessment of Case–control Studies with NOS									
	Selection				Comparability		Exposure			NOS QS
	1	2	3	4	5	6	7	8	9	
LIM, S	*		*	*	*		*	*		6
Zhang, Z	*	*		*	*	*	*	*		7
Rao, S B	*		*	*		*	*	*	*	7
Pennington, Z	*		*			*	*		*	5
Kim, J	*	*	*	*	*	*	*	*		8
Seki, T	*	*		*	*		*	*		6
Kim, B D	*		*	*		*	*		*	6
Miller, E M	*	*		*		*	*	*	*	7
De la Garza-Ramos, R	*	*		*	*		*		*	6
Wang, S	*	*	*	*	*	*		*	*	8
White, SJW	*		*	*		*	*	*		6

### Statistical analysis

Stata version 14.0 (Stata Corp LP, College Station, Texas) was used to synthesize, summarize, and evaluate the data. The collected data were tested for heterogeneity and the combined OR value and 95%CI were calculated. To determine heterogeneity across the studies, the I^2^ Higgins (0–100%) was adopted. The fixed-effect model was used for meta-analysis when the heterogeneity statistic I^2^ is less than 50%. In the meanwhile, the random-effect model was applied when the heterogeneity statistic I^2^ is greater than or equal to 50%. Funnel plot was used to analyze potential publication bias when the number of articles included was more than 5. Sensitivity analysis was used to test the stability of meta-analysis results: (1) comparison of results between random effect model and fixed effect model; (2) When the number of included literatures is more than 5, the points with significant deviation from 95%CI in the funnel chart are excluded for meta-analysis, and the results are compared with those when all the literatures are included. The *p* value for statistical significance was set at < 0.05.

## Results

### Study selection

**Fig. 1:**
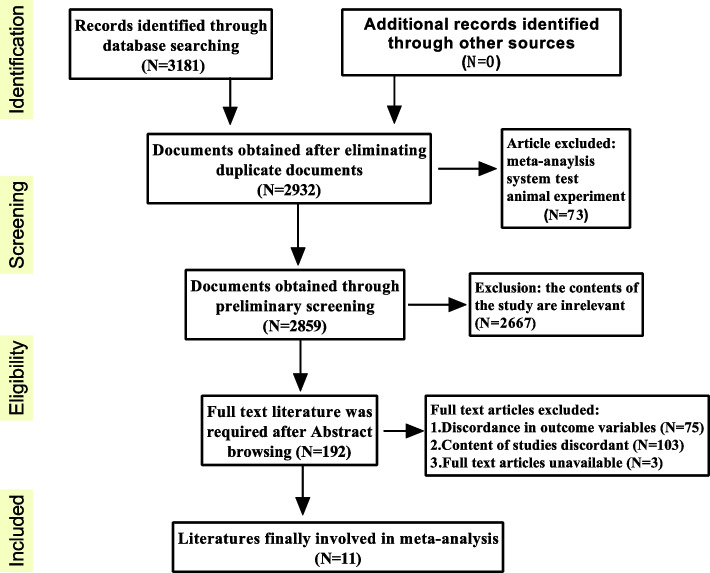
Flow chart of the searched, identified and included studies for meta-analysis

According to the search terms of the literature, a total of 3181 relevant articles were initially identified. Of those articles, 249 were duplicated in databases. After screening the remaining 2932 articles using titles and abstracts, most of the studies were excluded because they were not relevant to the objectives of this study (2667), case reports or meta-analysis and reviews (73). After reading the full text of the remaining 192 articles, a total of 181 were excluded due to the inability to obtain the full text (3), the outcome variables did not match (75), research content does not meet inclusion standards (103). Finally, 11 articles were included in this study for data extraction and meta-analysis (Fig. 1).

### Study characteristics

The eligible studies included 11 case–control study. The highest NOS score was 8 and the lowest was 5. A total of 1464 patients with non-superficial SSI after spinal surgery were included in the study. Among them, 1043 patients had deep-SSI, 157 patients had organ/space SSI, and 264 patients had deep-SSI or organ/space SSI. The basic characteristics and NOS scores of the included studies are shown in Table 2.

**Table 2:** Characteristics and quality evaluation of the included studies

Author, year	Study design	Date of data collection	Samples that meet the inclusion criteria (N)	Type of operation	d-SSI or organ/space SSI	risk factors	Statistical method	NOS scores
**LIM, S 2018 **[2]	retrospective study	2006–2011	111	Single level lumbar fusion surgery	organ/space SSI	1. age (> 70) 2. female 3. ASA > 2 4. operative time > 6 h	Logistic regression analysis	6
**Zhang, Z 2015 **[14]	retrospective study	2008–2013	99	Posterior thoracic and lumbar surgery	d-SSI	1. obesity 2. diabetes mellitus 3. number of surgical levels ≥ 3	Logistic regression analysis	7
**Rao, S B 2011 **[15]	Prospective study	2008.01–2008.12	57	Posterior spine fusion surgery	d-SSI	1. drainage time(per/day) 2.BMI 3.male	Logistic regression analysis	7
**Pennington, Z 2019 **[16]	retrospective study	2012–2018	38	Spinal surgery	d-SSI	1. drainage time(per/day) 2.BMI 3.hemoglobin	Logistic regression analysis	5
**Kim, J 2022 **[17]	retrospective study	2012–2018	767	Instrumented spinal fusion surgery	d-SSI	1. age > 60 2.male 3.rural residence 4.multiple approaches 5.cerebrovascular disease 6.peripheral vascular disease 7.chronic pulmonary disease 8.rheumatologic disease 9.liver disease 10.diabetes mellitus 11.hemiplegia or paraplegia 12.allogenous transfusion 13.use of systemic steroids > 2 weeks	Logistic regression analysis	8
**Seki, T 2013 **[18]	retrospective study	2007–2012	10	Spinal surgery	d-SSI	1. operative time 2. drainage time	Logistic regression analysis	6
**Kim, B D 2014 **[19]	retrospective study	2006–2011	unknown	Single-level lumbar fusion surgery	organ/space SSI	1. operative time > 5 h	Logistic regression analysis	6
**Miller, E M 2022 **[20]	retrospective cohort study	2009–2019	unknown	Anterior lumbar interbody fusion surgery	organ/space SSI	1. BMI > 35	Logistic regression analysis	7
**De la Garza-Ramos, R 2016 **[21]	retrospective study	2006–2012	264	Surgery for degenerative spine disease	d-SSI or organ/space SSI	1. renal morbidity 2. hemato-oncological morbidity 3.chronic steroid use 4. diabetes mellitus 5.obesity 6. lengths of stay 7.operative time	Logistic regression analysis	6
**Wang, S 2022 **[22]	retrospective study	2013–2018	20	Posterior lumbar interbody fusion surgery	d-SSI	1. local use of vancomycin powder 2.BMI 3. drinking 4. urinary tract infections 5. diabetes mellitus 6. blood transfusions 7. number of surgical levels 8. postoperative drainage volume	Logistic regression analysis	8
**White, SJW 2019 **[23]	retrospective study	2008–2015	52	Elective anterior lumbar fusion surgery	d-SSI	1. steroid use	Logistic regression analysis	6

### Meta‑analysis

According to the research contents of the included literature and the number of references for each factor, five risk factors including diabetes mellitus, obesity, using steroids, drainage time and operative time were selected for meta-analysis.

### Operative time

**Fig. 2:**
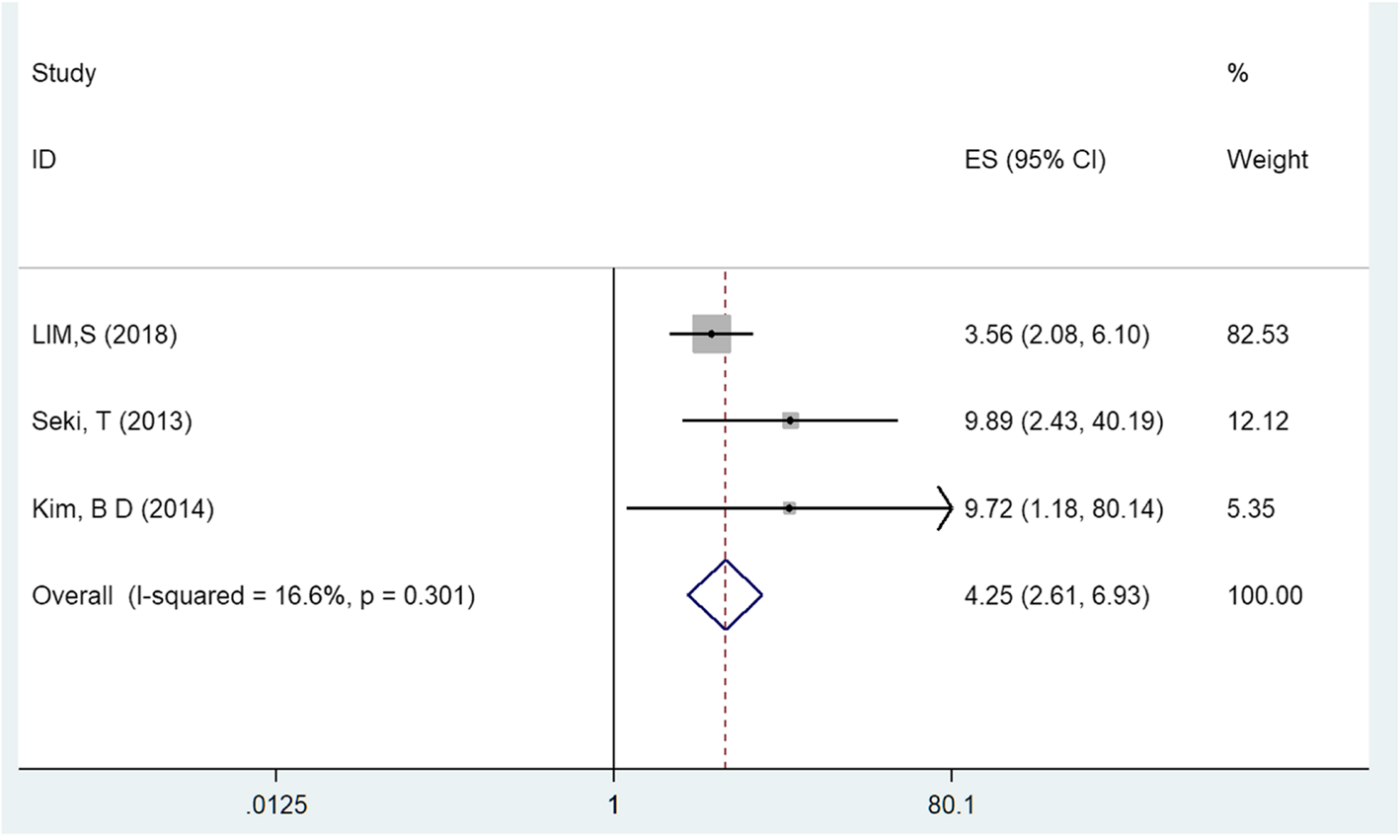
Multivariate analysis of operative time in a forest map

Three literatures reported operative time as a risk factor of postoperative non-superficial surgical site infections. There was no heterogeneity among these studies (I^2^ = 16.6%, *P* = 0.301). Meta-analysis of the three included studies using fixed effect model showed that operation time had a significant effect on the d-SSI or organ/space SSI[OR = 4.255, 95%CI (2.612,6.932), *P* < 0.05, Fig. 2] [2, 18, 19].

### Using steroids

**Fig. 3:**
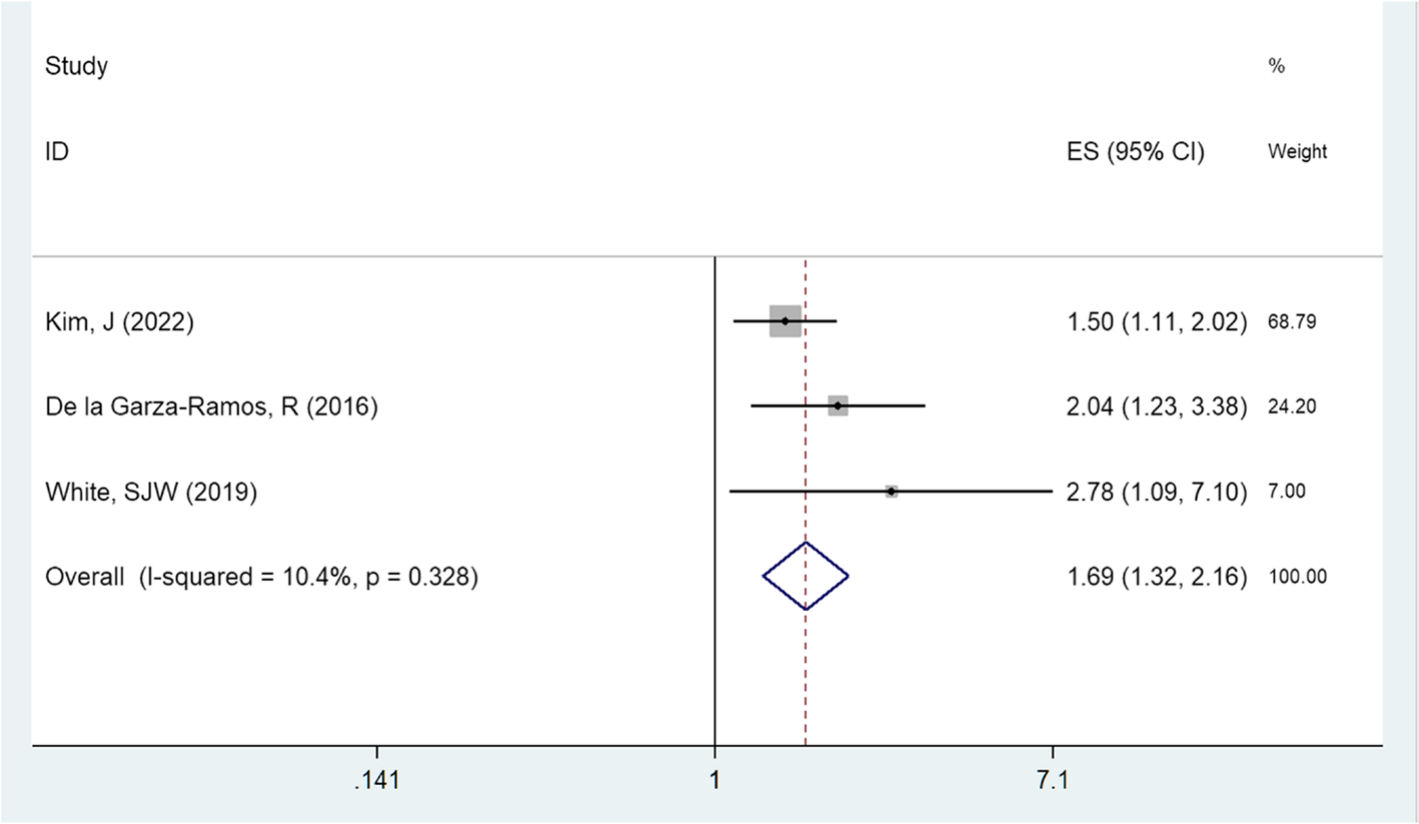
Subgroup analysis of using steroids in a forest map

Three studies reported the relationship between using steroids and non-superficial SSI [17, 21, 23]. There was no heterogeneity among these studies (I^2^ = 10.4%, *P* = 0.328), therefore the fixed effect model was used to statistically analyze the data. The results showed that using steroids was a risk factor affecting non-superficial SSI [OR = 1.687, 95%CI (1.317,2.162), *P* < 0.05, Fig. 3].

### Drainage time

**Fig. 4:**
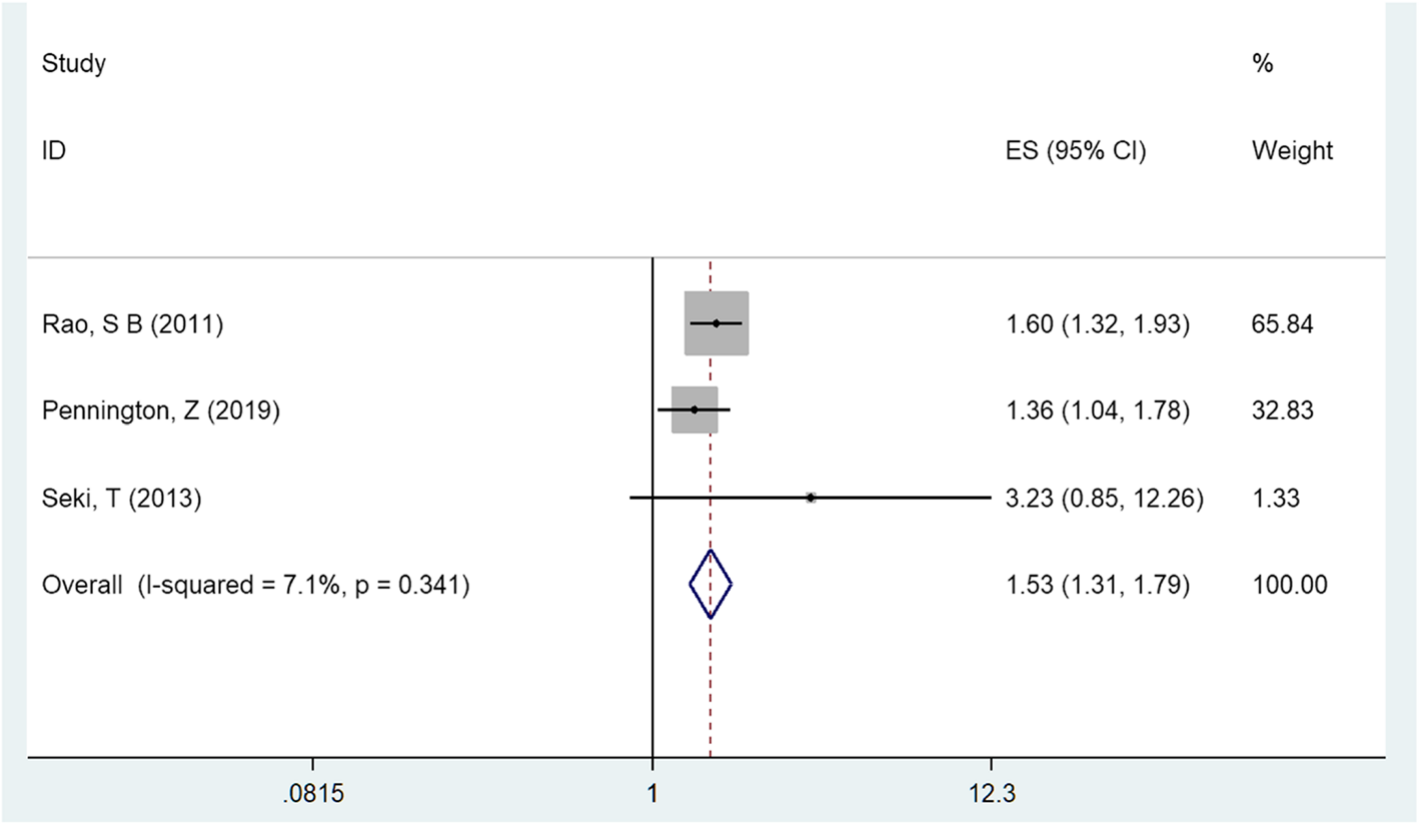
Multivariate analysis of drainage time in a forest map

Meta-analysis of the three included studies using fixed effect model showed that drainage time had a significant effect on the d-SSI or organ/space SSI [OR = 1.531, 95%CI (1.313,1.786), *P* < 0.05, Fig. 4] and no heterogeneity was observed between the three studies (I^2^ = 7.1%, *P*= 0.341) [15, 16, 18].

### Diabetes mellitus

**Fig. 5:**
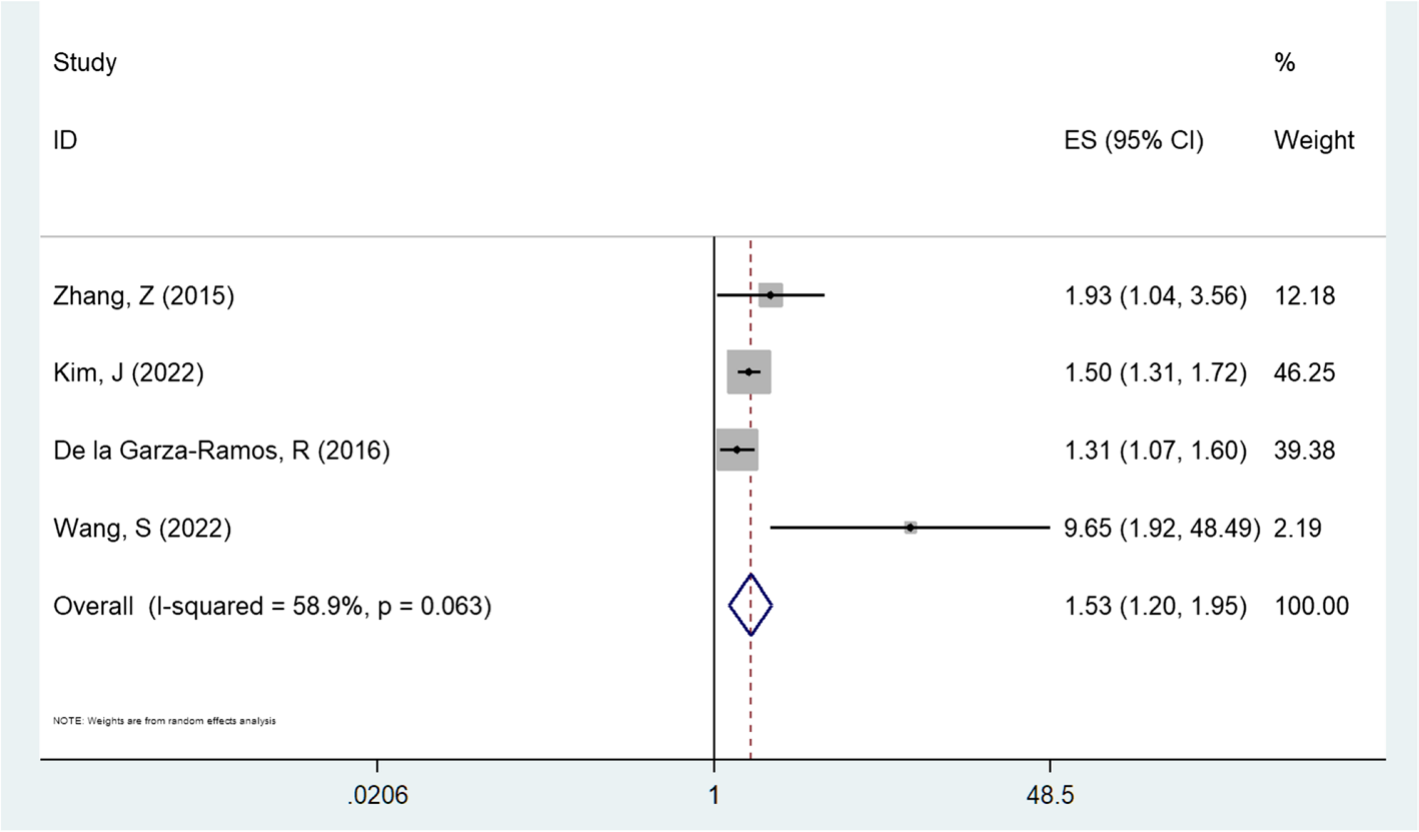
Multivariate analysis of diabetes mellitus in a forest map

Taking diabetes mellitus as an independent factor, the results of meta-analysis using a random effect model showed that there was correlation between the diabetes mellitus and the non-superficial surgical site infections [combined OR values = 1.527, 95%CI (1.196, 1.949), *P* = 0.001, Fig. 5]. In addition, slight heterogeneity was found among the studies (I2 = 58.9%, *P* = 0.063) [14, 17, 21, 22].

### Obesity

**Fig. 6:**
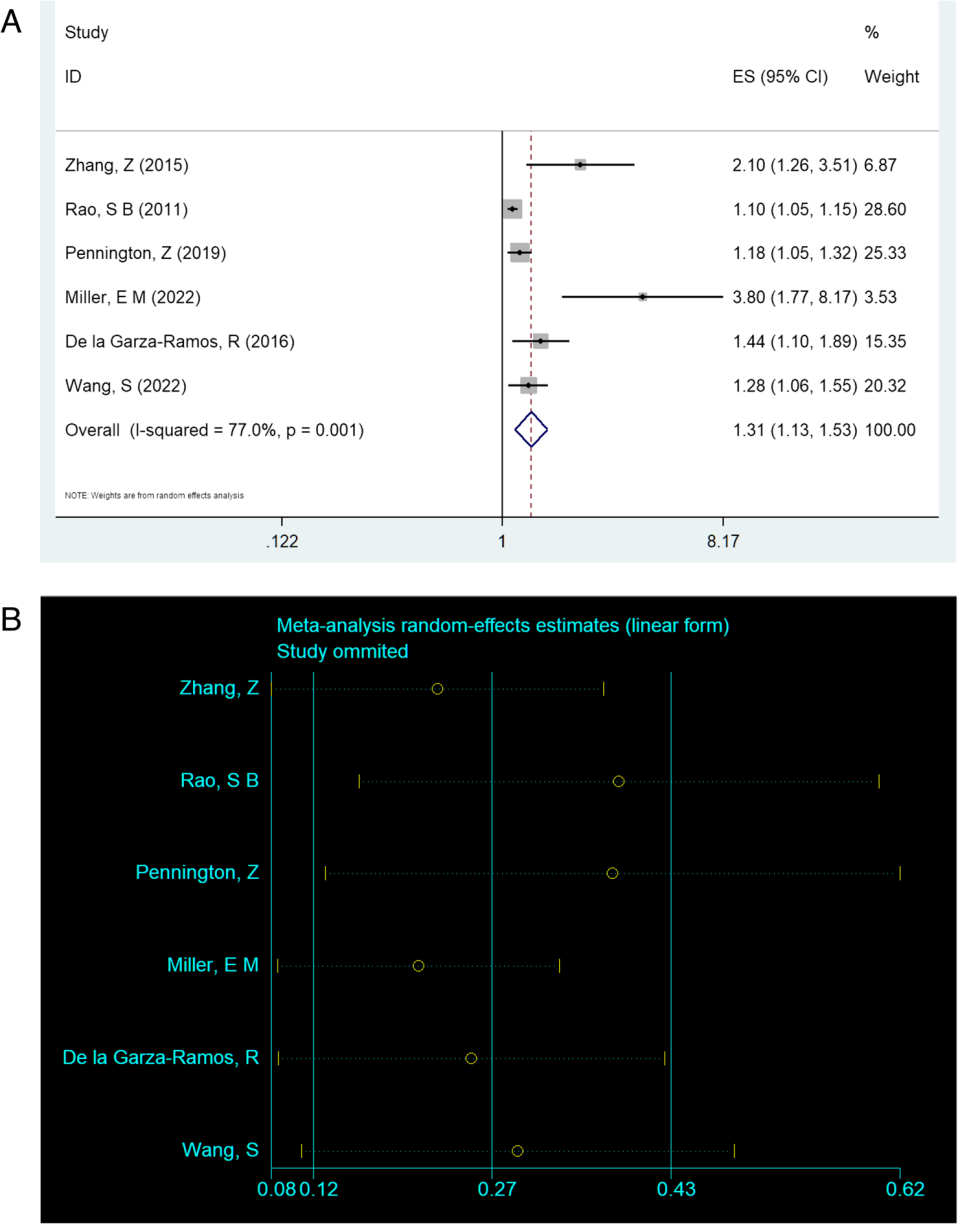
**A** Multivariate analysis of obesity in a forest map. **B** Sensitivity analysis of literatures was conducted by adopting the one-by-one elimination method

Six studies reported that the OR value between obesity and non-superficial surgical site infections was 1.314[95%CI (1.128, 1.532), *P* = 0.000], and there was great heterogeneity between studies (I^2^ = 77.0% %, *P*= 0.001) [14–16, 20–22]. The forest plot of obesity and the non-superficial SSI is also shown in Fig. 6A. Sensitivity analysis of literatures was conducted by adopting the one-by-one elimination method, and no studies with a large impact on heterogeneity were found (Fig. 6B).

### Publication bias analysis

**Fig. 7:**
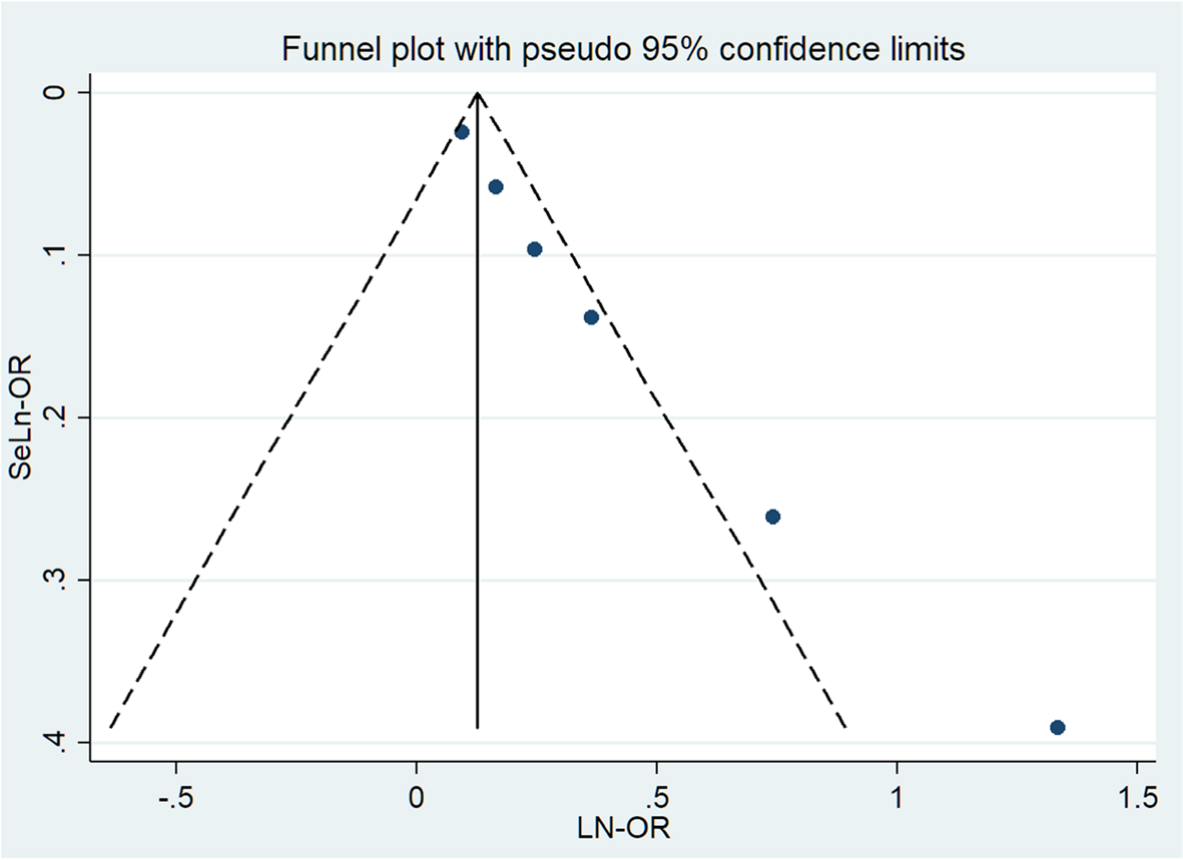
Funnel plot of obesity

Taking diabetes mellitus, obesity, steroids, drainage time and operative time as indicators to detect publication bias, the Egger's and Begg's test results are as follows: 1) diabetes mellitus (0.248, 0.308); 2) obesity (0.000, 0.009); 3) steroids (0.148, 0.296); 4) drainage time (0.638, 1.000); 5) operative time (0.210, 1.000). The above test results are all *P* > 0.05 except obesity, indicating that there is little possibility of publication bias in diabetes mellitus, steroids, drainage time, operative time. However, the funnel plot of obesity is not symmetrical, indicating that operative time, as one of the risk factors, has publication bias (Fig. 7).

## Discussion

According to the definition of CDC, there are three types of SSI. Superficial SSI may cause local symptoms such as redness, swelling, heat and pain in the early stage of infection, which is characterized by mild symptoms and good prognosis. However, deep SSI and organ/space SSI are not easily identified and result in severe symptoms and poor clinical outcomes. Therefore, we explore the risk factors that may lead to non-superficial SSI, in order to guide clinicians to conduct more precise treatment.

This meta-analysis systematically collected relevant studies on risk factors of deep surgical site infections and organ/space surgical site infections after spine surgery. Various literatures had reported the risk factors of postoperative SSI [5, 24]. However, we were surprised to find that there was no meta-analysis of postoperative d-SSI or organ/space SSI, which could result in a worse prognosis outcome in SSI.

As a risk factor of postoperative non-superficial SSI, diabetes mellitus will lead to the decline of body immunity, and then cause SSI [25–27]. Impairment of protein metabolism in patients with poor long-term glycemic control reduces the production of important substances such as immunoglobulin complement, antibodies, and enzymes. Defects that tend to have multiple defense functions simultaneously are susceptible to infection and difficult to control. Secondly, it may results in increasing the probability of microvascular disease and blocking blood flow in wound area [28]. In addition, due to the fact that high glucose blood is a good growth environment for bacteria and fungi, it is easy to cause breeding and propagation, leading to surgical site infection. To explore the reason for slight heterogeneity of diabetes mellitus, we found that the OR value obtained from Dr. Wang was relatively high. It may be caused by small sample size from Dr. Wang 's article (*N* = 20), which is less than the other three articles (99; 767; 264 respectively).

Obesity is associated with alterations in peripheral blood lymphocyte cytokine expression, according to O'Rourke RW et al. The adipose tissue participates actively in inflammation and immunity maybe the mechanisms that predispose obese patients to infection. When obese, there will be an increase in local tissue trauma as a result of retraction, longer operative times, and a disturbance of the body's homeostatic balance [29]. BMI, BMI > 35 and obesity were all included in this meta-analysis study. Subcutaneous fat thickness has also been considered as a risk factor for postoperative SSI, but it is still controversial [30–32]. Due to the thicker subcutaneous fat in obese patients, local fat necrosis will occur after surgical operation, resulting in increasing probability of d-SSI or organ/space SSI [30]. Some authors suggested that BMI > 30 was the risk factor for postoperative non-superficial SSI. Others thought BMI > 35 may lead to an increased probability of non-superficial SSI [20]. Even some authors did not propose specific definitions for obesity in their article [14, 21]. Therefore, for the moderate heterogeneity of obesity, we speculate that it may be due to different definitions of obesity.

Steroids are effective in the treatment of autoimmune diseases because they reduce the function of the immune system. Patients taking steroids are not only more likely to get infected, but also more likely to develop serious or unusual infections [33]. In this meta-analysis, we observed that the incidence of non-superficial SSI was 1.687 times higher in patients who received steroids than in those who did not.

Drainage tube may increase the risk of SSI by causing local tissue inflammation or bacterial retrograde infection [15, 34]. The longer the drainage tube stays, the higher the patient's risk of postoperative wound infection. In addition, insufficient drainage may lead to hematoma and infection. However, prolonged drainage tube indwelling time is also a risk factor for postoperative non-superficial SSI. When the drainage tube should be removed is still controversial [35, 36].

Due to long-term surgery, patients are often accompanied by large wound areas, bleeding, and local hematoma, which reduces systemic resistance, disruption of the normal vasculature of the surrounding tissues and increases the rate of incision infection. As the operation time increases, the exposure time of the incision to the air increases accordingly. The patient's resistance to bacterial infection also decreases during anesthesia, which is a prerequisite for surgery [19]. In addition, longer operative time predisposes incisions to tissue desiccation that may also increase the probability of contamination [37, 38]. All above reasons make prolonging surgical time a critical risk factor for non-superficial SSI. Compared with other risk factors, the operation time directly affects the contact time between external bacteria and the surgical site incision. In this study, we draw a conclusion that operation time is the most critical factor leading to postoperative non-superficial SSI. Therefore, for surgeons, improving surgical proficiency and shortening operation time are effective measures to reduce postoperative SSI.

To our knowledge, this is the first meta-analysis of risk factors for non-superficial surgical site infection after spinal surgery. This study draws the conclusion that diabetes mellitus, obesity, using steroids, drainage time and operative time are risk factors for non-superficial SSI following spinal surgery.

## Limitations

Due to strict exclusion criteria, some potential risk factors (such as ASA > 2, Allogeneic blood transfusion, length of stay and so on) were not included, so more original studies are needed.

## Conclusion

At present, previous studies on the factors affecting the postoperative non-superficial SSI are controversial. Therefore, this meta-analysis was conducted. The results showed that diabetes mellitus, obesity, using steroids, drainage time and operative time are risk factors for non-superficial SSI following spinal surgery. In this study, operative time is the most important risk factor resulting in postoperative d-SSI and organ/space SSI.

## Supplementary Information

**Additional file 1.** Exact retrieval strategy.

## Data Availability

The datasets of the current study are available from the corresponding author upon reasonable request.

## Abbreviations

SSI: Surgical site infection
d-SSI: Deep surgical site infection
NOS: The Newcastle–Ottawa Scale
CDC: The Centers for Disease Control and Prevention
BMI: Body Mass Index
ASA: American Society of Anesthesiologists
