# Supplementary material for: A meta-analysis of risk factors for non-superficial surgical site infection following spinal surgery
Source: BMC Surg. 2023 May 16;23:129. doi: 10.1186/s12893-023-02026-2 (PMC10186697; doi:10.1186/s12893-023-02026-2)
Supplement: Supplementary file 1 — Additional file 1. Exact retrieval strategy. [file 12893_2023_2026_MOESM1_ESM.docx]

Exact retrieval strategy:

((spin*[Title/Abstract]) AND ((((((infections[Title/Abstract]) OR (Infection[Title/Abstract] AND Infestation[Title/Abstract])) OR (Infestation[Title/Abstract] AND Infection[Title/Abstract])) OR (Infections[Title/Abstract] AND Infestations[Title/Abstract])) OR (Infestations[Title/Abstract] AND Infections[Title/Abstract])) OR (Infection[Title/Abstract]))) AND ((((((((((((((((risk factors[Title/Abstract]) OR (Factor, Risk[Title/Abstract])) OR (Risk Factor[Title/Abstract])) OR (Social Risk Factors[Title/Abstract])) OR (Factor, Social Risk[Title/Abstract])) OR (Factors, Social Risk[Title/Abstract])) OR (Risk Factor, Social[Title/Abstract])) OR (Risk Factors, Social[Title/Abstract])) OR (Social Risk Factor[Title/Abstract])) OR (Health Correlates[Title/Abstract])) OR (Correlates, Health[Title/Abstract])) OR (Population at Risk[Title/Abstract])) OR (Populations at Risk[Title/Abstract])) OR (Risk Scores[Title/Abstract])) OR (Risk Score[Title/Abstract])) OR (Score, Risk[Title/Abstract]))
